# Supplementary material for: Empirical methods for controlling false positives and estimating confidence in ChIP-Seq peaks
Source: BMC Bioinformatics. 2008 Dec 5;9:523. doi: 10.1186/1471-2105-9-523 (PMC2628906; doi:10.1186/1471-2105-9-523)
Supplement: Additional File 1 — A variety of html documents from the USeq web site detailing the available applications, their best usage, output file type descriptions, command line menus, etc. [file 1471-2105-9-523-S1.zip › USeqUserGuides/JavaDoc.zip!/JavaDoc/util/bio/annotation/GeneRep.html]

GeneRep


|  |  |  |
| --- | --- | --- |
| **Overview** \| **Package** \| **Class** \| **Use** \| **Tree** \| **Deprecated** \| **Index** \| **Help** | |  |
| **PREV CLASS**   **NEXT CLASS** | **FRAMES**    **NO FRAMES**     **All Classes** |  |
| SUMMARY: NESTED \| FIELD \| CONSTR \| METHOD | DETAIL: FIELD \| CONSTR \| METHOD |  |


---


## util.bio.annotation Class GeneRep

```
java.lang.Object
  util.bio.annotation.GeneRep
```

**All Implemented Interfaces:**: java.io.Serializable

---

``` public class GeneRep extends java.lang.Object implements java.io.Serializable ```

A GeneRep resentation contains a genomic sequence designating a gene broken into various segments.
This takes the most conservative approach. Any annotation that says something is coding is shown as coding.
Exons are maximized, introns and UTRs minimalized (actually UTR refers to RNA, not genomic).
Different translations/transcriptions will give various versions of these segments.
There is no orientation for these features! Use the GeneGroup to get the real start/stop and thus the orientation
and strand.

**See Also:**: Serialized Form

---

**Constructor Summary**

`GeneRep(TransGroup[] transGrps,
GeneGroup geneGrpRef)`


| **Method Summary** | |
| --- | --- |
| `java.util.ArrayList` | `getCodingSegments()`             Conservative estimate of coding segments. |
| `int[]` | `getFivePrimeNonCodingRegion()`             Conservative estimate returning smallest non coding region. |
| `GeneGroup` | `getGeneGrp()` |
| `java.util.ArrayList` | `getNonCodingSegments()`             Conservative estimate of non coding segments between coding exons. |
| `int` | `getStartATGPosition()`             Conservative estimate of the start ATG position, from the longest transcript |
| `int[]` | `getThreePrimeNonCodingRegion()`             Conservative estimate returning smallest non coding region. |
| `boolean` | `isTranslationFlag()` |
| `java.lang.String` | `toString()` |

**Methods inherited from class java.lang.Object**

`equals, getClass, hashCode, notify, notifyAll, wait, wait, wait`

**Constructor Detail**

### GeneRep

```
public GeneRep(TransGroup[] transGrps,
               GeneGroup geneGrpRef)
```


**Method Detail**

### toString

```
public java.lang.String toString()
```

:   **Overrides:**: `toString` in class `java.lang.Object`

---


### getGeneGrp

```
public GeneGroup getGeneGrp()
```

---


### isTranslationFlag

```
public boolean isTranslationFlag()
```

---


### getCodingSegments

```
public java.util.ArrayList getCodingSegments()
```

:   Conservative estimate of coding segments. Returns biggest from all translations.

---


### getNonCodingSegments

```
public java.util.ArrayList getNonCodingSegments()
```

:   Conservative estimate of non coding segments between coding exons. Not introns!

---


### getFivePrimeNonCodingRegion

```
public int[] getFivePrimeNonCodingRegion()
```

:   Conservative estimate returning smallest non coding region. Not the UTR!

---


### getThreePrimeNonCodingRegion

```
public int[] getThreePrimeNonCodingRegion()
```

:   Conservative estimate returning smallest non coding region. Not the UTR!

---


### getStartATGPosition

```
public int getStartATGPosition()
```

:   Conservative estimate of the start ATG position, from the longest transcript


---


|  |  |  |
| --- | --- | --- |
| **Overview** \| **Package** \| **Class** \| **Use** \| **Tree** \| **Deprecated** \| **Index** \| **Help** | |  |
| **PREV CLASS**   **NEXT CLASS** | **FRAMES**    **NO FRAMES**     **All Classes** |  |
| SUMMARY: NESTED \| FIELD \| CONSTR \| METHOD | DETAIL: FIELD \| CONSTR \| METHOD |  |


---
